# Neuroregeneration in 6‐OHDA‐Lesioned Adult Zebrafish Involves Glia‐Guided Migration of Dopaminergic Neurons from Olfactory Bulb and Telencephalon to Dienchephalon

**DOI:** 10.1002/alz70855_107621

**Published:** 2025-12-25

**Authors:** Naemah Md Hamzah, Siong Meng Lim, Fei Tieng Lim, Mitra Amiri Khabooshan, Jan Kaslin, Abu Bakar Abdul Majeed, Kalavathy Ramasamy

**Affiliations:** ^1^ Faculty of Pharmacy, Universiti Teknologi MARA, Puncak Alam, Selangor, Malaysia; ^2^ Collaborative Drug Discovery Research (CDDR) Group, Faculty of Pharmacy, Universiti Teknologi MARA (UiTM), Puncak Alam, Selangor, Malaysia; ^3^ Australian Regenerative Medicine Institute, Clayton, VIC, Australia; ^4^ Brain Degeneration and Therapeutics Group, Faculty of Pharmacy, Universiti Teknologi MARA (UiTM), Puncak Alam, Selangor, Malaysia

## Abstract

**Background:**

Neurodegenerative diseases are the fastest‐growing disorders. Current medications, however, offering only symptomatic relief but fail to halt or reverse their progression. This raises the need for novel therapeutic strategies, such as promoting brain self‐repair. The process is inefficient in mammals, making mammalian‐based models unsuited for understanding of neuroregeneration. As such, adult zebrafish is greatly favoured for its close brain homology with human and neuronal self‐renewal capability. Our previous findings suggested that the lost dopaminergic neurons (DpN) in the ventral diencephalon (vDn; lesion site) of 6‐OHDA‐lesioned adult zebrafish could likely be restored by newly regenerated cells that migrated from the olfactory bulb (OB) and the telencephalon (Tel). This study went on to examine the potential involvement of glial cells in facilitating the migration of the newly regenerated cells.

**Method:**

The whole‐brain imaging was used to investigate glial cell expression at day‐14 post‐6‐OHDA lesion (i.e., a timepoint associated with neuronal migration). The markers of interest included gfap (glial fibrillary acidic protein), olig2 (oligodendrocyte transcription factor 2), and glula (glutamine synthetase a). IHC was performed on the marker with the most significant expression to assess specific glial cell expression and their localisation at the peak migration timepoint (day‐14) and earlier timepoints (day‐5 and day‐7).

**Result:**

A significantly (*p* < 0.05) higher number of gfap‐immunoreactive cells was detected in the vDn and adjacent Tel at day‐14 post‐lesion when compared to other regions. This is in line with IHC analysis which also revealed the presence of glial cells in the preoptic area (POA), a region near the lesion site.

**Conclusion:**

These findings confirmed the presence of glial cells near the POA and vDn lesion site at day‐14 post‐6‐OHDA lesion, suggesting their potential role in guiding neuronal migration. This warrants further investigations into the roles of specific glial cells in facilitating DpN regeneration.

